# CircWalk: a novel approach to predict CircRNA-disease association based on heterogeneous network representation learning

**DOI:** 10.1186/s12859-022-04883-9

**Published:** 2022-08-11

**Authors:** Morteza Kouhsar, Esra Kashaninia, Behnam Mardani, Hamid R. Rabiee

**Affiliations:** 1 BCB Lab, Department of Computer Engineering, Sharif University of Technology, Tehran, Iran; 2 Department of Computer Science and Information Technology, Institute for Advanced Studies in Basic Sciences (IASBS), Zanjan, Iran

**Keywords:** circRNA, ceRNA, Disease, Biological network, Representation learning

## Abstract

**Background:**

Several types of RNA in the cell are usually involved in biological processes with multiple functions. Coding RNAs code for proteins while non-coding RNAs regulate gene expression. Some single-strand RNAs can create a circular shape via the back splicing process and convert into a new type called circular RNA (circRNA). circRNAs are among the essential non-coding RNAs in the cell that involve multiple disorders. One of the critical functions of circRNAs is to regulate the expression of other genes through sponging micro RNAs (miRNAs) in diseases. This mechanism, known as the competing endogenous RNA (ceRNA) hypothesis, and additional information obtained from biological datasets can be used by computational approaches to predict novel associations between disease and circRNAs.

**Results:**

We applied multiple classifiers to validate the extracted features from the heterogeneous network and selected the most appropriate one based on some evaluation criteria. Then, the XGBoost is utilized in our pipeline to generate a novel approach, called CircWalk, to predict CircRNA-Disease associations. Our results demonstrate that CircWalk has reasonable accuracy and AUC compared with other state-of-the-art algorithms. We also use CircWalk to predict novel circRNAs associated with lung, gastric, and colorectal cancers as a case study. The results show that our approach can accurately detect novel circRNAs related to these diseases.

**Conclusions:**

Considering the ceRNA hypothesis, we integrate multiple resources to construct a heterogeneous network from circRNAs, mRNAs, miRNAs, and diseases. Next, the DeepWalk algorithm is applied to the network to extract feature vectors for circRNAs and diseases. The extracted features are used to learn a classifier and generate a model to predict novel CircRNA-Disease associations. Our approach uses the concept of the ceRNA hypothesis and the miRNA sponge effect of circRNAs to predict their associations with diseases. Our results show that this outlook could help identify CircRNA-Disease associations more accurately.

**Supplementary Information:**

The online version contains supplementary material available at 10.1186/s12859-022-04883-9.

## Background

Non-coding RNAs are essential to cell players who manipulate and control many biological processes. About 80–90% of human cell transcripts are non-protein-coding [1]. There are multiple types of non-coding RNAs, and each of them has specific functions in the complex system of gene regulation. One of the essential non-coding RNAs that researchers have recently noticed is Circular RNAs (circRNAs). circRNAs created from other transcripts through a non-canonical splicing event called back splicing. In this process, the transcript's 5′ and 3′ splice sites bind together and reconstruct a circular shape called circRNA [2]. This circular structure makes the circRNAs more stable than other RNAs [3, 4] and makes them attractive as a biomarker in complex diseases [5, 6].

Multiple functions have been identified for the circRNAs in the cell [7, 8]. They can act as enhancers for the role of other proteins or as scaffolds to mediate complex formation for some enzymes [2]. circRNAs also regulate the RNA Binding Proteins (RBP) by decoying them [2]. One of the most critical functions for circRNAs is trapping miRNAs based on their sequence and miRNA response elements (MREs) [9]. By sponging shared miRNAs, circRNAs can regulate the expression of coding RNAs [8]. This mechanism is known as the competing endogenous RNA (ceRNA) hypothesis [10], which is involved in multiple complex diseases such as cancer [11].

circRNAs are involved in many human diseases based on previous research [5, 12, 13]. For instance, circRNA Cdr1as affect insulin secretion in the pancreatic islet cells via decoying miR-7 miRNA. Consequently, this circRNA is a therapeutic target for diabetes [14]. hsa_circ_0054633 is another circRNA that is overexpressed in patients with type 2 Diabetes Mellitus [15]. Recently, two other circRNAs (hsa_circ_0063425 and hsa_circ_0056891) have been introduced as novel biomarkers to predict type 2 Diabetes Mellitus in the early stages [16]. In cardiovascular diseases, circRNA HRCR absorbs miR-233 and prevents heart failure [17]. circFndc3b is another critical cardio-related circRNA that has recently been detected. It is involved in cardiac repair pathways [18]. Alzheimer's disease (AD) is another disease in which the role of proteins has been proven [19]. For instance, a circular RNA created from the IGF2R transcript is associated with AD pathology [20]. Many circRNAs have also been involved in multiple cancer types [21]. For example, in glioma, circRNA 0001445 promotes tumor progression through the miRNA-127-5p/SNX5 signaling pathway [22]. hsa_circ_0062019 promotes prostate cancer cell proliferation, migration, and invasion through upregulating HMGA2 by decoying miR-195-5p [23]. Many other studies demonstrate the role of circRNAs in multiple cancer types such as thyroid, gastric, bladder, breast, and colon cancer [24–28].

Developing high-throughput technology such as RNA Sequencing (RNA-Seq) and public databases to store them has provided a valuable resource for researchers to create novel computational algorithms to mine the biological data. In circRNA-related studies, computational algorithms such as deep learning and machine learning-based methods can help predict more accurate CircRNA-Disease associations and deeply understand disease mechanisms. Many computational approaches have been developed to predict CircRNA-Disease associations in recent years. These approaches can be categorized into two main groups: network algorithm-based models and machine learning-based models [8]. Generally, network-based algorithms combine multiple resources to generate a circRNA-Disease association network and predict novel interactions. For example, IBNPKATZ integrates the bipartite networks from known circRNA-Disease associations and circRNAs similarities and uses the KATZ measure to find novel circRNA-Disease pairs [29]. Ge et al. developed a network approach based on locality-constrained linear coding and label propagation [30]. One of the most recent algorithms has been introduced by Lei et al. [31]. They reconstructed a heterogeneous network based on known circRNAs and disease relationships, circRNA-circRNA, and disease-disease similarities. After that, a novel weighted biased meta-structure search algorithm was applied to the network to predict CircRNA-Disease associations. A heterogeneous network was reconstructed in a similar approach by Zhang et al. [32]. They used multiple resources to create circRNA and disease similarity networks. In their novel algorithm, entitled PCD_MVMF, the metapath2vec++ method was applied on meta paths in the heterogeneous network. Then the matrix factorization algorithm was used to predict the novel association between circRNAs and diseases. A combination of deep learning and matrix factorization methods was also used in another study. The DMFCDA (Deep Matrix Factorization CircRNA-Disease Association) algorithm was developed based on this approach [33]. Lu et al. developed a deep learning-based algorithm called CDASOR to predict CircRNA-Disease associations based on sequence and ontology representations with convolutional and recurrent neural networks [34]. In another study, Deng et al. proposed the KATZCPDA algorithm based on a previously developed algorithm (KATZ) [35, 36]. The KATZCPDA algorithm integrated circRNA-protein and protein-disease association data with circRNA similarity and disease similarity data to reconstruct a heterogeneous network. Subsequently, a KATZ measure [35] was applied to extract unknown CircRNA-Disease associations by measuring the similarities between circRNAs and diseases [36].

Generally, in many computational approaches to predicting CircRNA-Disease associations, interaction data between circRNAs and diseases from multiple resources integrated with circRNA similarity and disease similarity data to reconstruct a heterogeneous network in which the association between circRNAs and disease is hidden and should be mine. The basic concept in these methods is that similar circRNAs may be associated with similar disorders. In these approaches, more accurate data integration causes more accurate results. Similarly, this article proposed a novel algorithm called CircWalk to accurately extract potential CircRNA-Disease associations from a heterogeneous network based on a network representation algorithm. One of the essential circRNAs functions is acting as a miRNA sponge based on the ceRNA hypothesis. Many circRNAs are associated with diseases based on this mentioned mechanism. Our proposed method tried to integrate data based on the ceRNA hypothesis to reconstruct the heterogeneous network. Our results demonstrated that this strategy could predict more accurate CircRNA-Disease associations compared with other algorithms.

## Methods

**Fig. 1:**
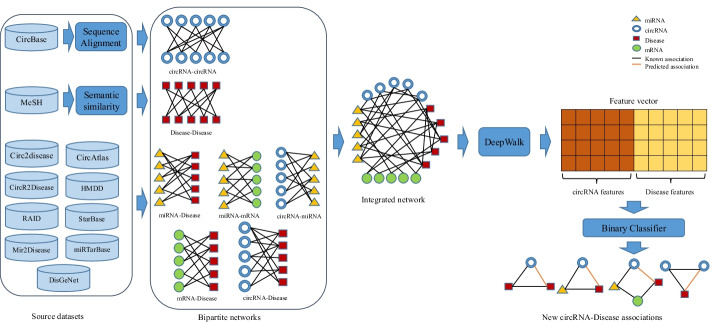
The overall workflow of the proposed algorithm

Our approach consists of three stages: In the first step, we merged data from multiple sources to reconstruct an informative heterogeneous network (Network reconstruction step). Next, we used the DeepWalk [37] algorithm to convert each circRNA and disease in this graph to a feature vector (Feature extraction step). At this stage, we have two feature vectors and a label (0 for unrelated and 1 for related pairs) for each CircRNA-Disease pair. We then train a classifier on this labeled dataset to create a model to predict CircRNA-Disease relationships accurately. Figure 1 shows the overall process of our algorithm.

### Network reconstruction

We merged multiple bipartite networks extracted from multiple experimentally validated databases to reconstruct a heterogeneous network. Seven types of bipartite networks (Fig. 1) were combined based on their common nodes (genes and diseases). One of the critical points in this step is to unify the genes and disease identifiers in all the networks before merging. Different disease datasets may use various names for the same disease, e.g., “hepatocellular cancer” and “hepatocellular carcinoma”. Therefore, we checked and unified the disease names in all bipartite networks. Similarly, circRNAs have multiple notations in various datasets. To avoid duplication, we used the CircBase dataset [38] as a reference to unify circRNA identifiers in our bipartite networks (the circRNAs that were not specified in CircBase were filtered out from the data). The mRNA and miRNA identifiers were identical in all data sources and didn’t need to be unified. Finally, we generated a heterogeneous network in which nodes represented circRNA, mRNA, miRNA, and disease, and the edges represented their relationships based on the bipartite networks extracted from the source databases. Based on the ceRNA hypothesis and the sponge effect of circRNAs, a circRNA can indirectly influence a disease. Therefore, adding mRNAs and miRNAs to this network can improve the prediction of indirect circRNA-Disease associations.

#### CircRNA-Disease

The data in Circ2Disease [39], CircR2Disease [40], CTD [41], and CircAtlas [42] were merged to generate CircRNA-Disease interactions.

#### circRNA-circRNA

We calculated the alignment scores between every two circRNAs in our data and regarded them as a similarity measure among circRNAs. Next, we set the average score of all pairs as a cutoff threshold. After that, the circRNA pairs whose similarity score was more significant than this threshold was considered circRNA-circRNA networks for further analysis. Human circRNA sequence data were downloaded from the CircBase database [38], and built-in functions calculated the similarity scores from the BioPython package [43].

#### circRNA-miRNA

We extracted the circRNA-miRNA interaction data by combining the pairs from experimentally validated data in RAID [44] and StarBase [45].

#### miRNA-disease

The experimentally validated data in Circ2Disease [39], HMDD [46], and Mir2Disease [47] were used to generate miRNA-disease interactions.

#### miRNA-mRNA

miRTarbase [48], Circ2Disease [39], and StarBase [45] were used to extract miRNA-mRNA bipartite network.

#### mRNA-disease

The experimentally validated data in DisGeNet [49] was used to obtain mRNA-disease associations.

#### Disease-disease

We use the tree structure of diseases in the MeSH [50] database for the disease-disease similarity network. We calculate the semantic similarity between each pair of diseases in our data, and all the similarities above than specific threshold (0.8) were considered disease-disease pairs. The method proposed by Wang et al. [51] in the pyMeSHSim python library [52] was used to calculate semantic similarity.

### Feature extraction

Given $G = \left( {V,E} \right)$ as a network in which $V = \left\{ {v_{1} ,v_{2} ,..v_{n} } \right\}$ is a set of nodes (RNAs and diseases) and $E = \left\{ {\left( {u,v} \right){|}u, v \in V} \right\}$ is a set of edges (interactions between the nodes). The goal of this step is to find a set of numeric feature vectors $X \in {\mathbb{R}}^{\left| V \right|*k}$ each of which represents a node in the network ($k$ is the size of each feature vector). The DeepWalk algorithm [37] solve this problem applying word2vec approach [53] on the random walks contains each node. DeepWalk solves an optimization problem (Eq. 1) to find maximum value of a mapping function ${\Phi }:v \in V \to {\mathbb{R}}^{\left| V \right|*k}$ for node $v_{i}$ in a random walk defined as $\left\{ {v_{i - w} ,.., v_{i} ,..,v_{i + w} } \right\}$.

1 $$\mathop {{\text{maximum}}}\limits_{{\Phi }} \mathop \prod \limits_{{\begin{array}{*{20}c} {j = i - w} \\ {j \ne i} \\ \end{array} }}^{i + w} {\text{P}}(v_{j} |{\Phi }\left( {v_{i} } \right))$$

We applied this algorithm on the network generated in the previous step and extracted a k-dimensional feature vector for each circRNA and Disease in the network. Since DeepWalk uses random walks (paths) on the graph to learn the embeddings of the nodes, we believe that adding new paths through mRNAs and miRNAs to the CircRNA-Disease graph can improve the performance of CircRNA-Disease associations prediction.

### Binary classification of CircRNA-disease Pairs

As a result of the previous step, we have a feature vector with a size of 2 k for each pair of CircRNA-Disease. Besides, there is a class label for each pair: 0 means the circRNA is unrelated to the disease, and 1 implies the circRNA is related to the disease. Consequently, we can define a dataset and learn a classifier to predict the label of each input pair. To this end, we generated a benchmark dataset (see the result section). We applied fivefold cross-validation based on multiple classifiers to evaluate the performance of extracted features from the heterogeneous network to predict the disease-related circRNAs. Six classification algorithms were used in this step: Support Vector Machine (SVM) [54], Logistic Regression (LR) [55], Random Forest (RF) [56], AdaBoost [57] with Random Forest base classifier (ABRF), XGBoost (XGB) [58], and Multilayer Perceptron (MP) [59]. All classifiers were applied to the data using the scikit-learn Python package [60] (For non-default classifier hyperparameters, see Additional file 1: Table S1).

## Results

### Evaluation metrics

The following evaluation metrics with fivefold cross-validation were used to evaluate the performance of our algorithm and compare it with some other state-of-the-art algorithms. For simplicity, we use the abbreviations TP, FP, TN, and FN for true positive, false positive, true negative, and false negative, respectively. The Area Under the receiver operating characteristic Curve (AUC) was the primary scoring metric we applied in comparing models against each other. To obtain this, we need to calculate the area under a plot with points whose x coefficients are the false-positive rates (FPR) of the model examined and whose y coefficients are the true positive rates (TPR) of that same model for different classification thresholds. TPR and FPR can be calculated based on Eqs. 2 and 3.

2 $$FPR = { }\frac{FP}{{FP + TN}}$$

3 $$TPR = { }\frac{TP}{{TP + FN}}$$

Accuracy (ACC) is the ratio of correctly classified samples to all samples and can be calculated based on Eq. 4.

4 $$Acc = \frac{TP + TN}{{TP + TN + FP + FN}}$$

Precision (Pre) is the ratio of true positive samples to all samples labeled as positive. We used Eq. 5 to calculate Pre for each algorithm.

5 $$Pre = \frac{TP}{{TP + FP}}$$

Sensitivity (Sen), also known as Recall, and Specificity (Spe) are the ratio of true positive samples and true negative samples to all ground truth, respectively. We calculated Sen and Spe based on Eqs. 3 and 6, respectively.

6 $$Spe = \frac{TN}{{TN + FP}}$$

The final evaluation metric is the F1 Score; the geometric mean of Pre and Sen. Equation 7 can be used to calculate this metric.

7 $$F1 = { }\frac{{2{ } \times Pre{ } \times Recall}}{Pre + Recall}$$

### Benchmark dataset

To evaluate our method, we need a labeled set of CircRNA-Disease pairs as a benchmark dataset, wherein the label is 1 if the pair are associated and 0 otherwise. The labels will later be used for supervised binary classification. To create the benchmark dataset, we adopted the approach in [61], in which an equal number of the positive samples were randomly selected from unknown pairs as negative samples. Our dataset has 575 known circRNA-Disease pairs reconstructed from 474 unique circRNAs and 64 unique diseases. Hence, there are 474 × 64 = 30,336 possible CircRNA-Disease combinations, 474 × 64 − 575 = 29,761 of which are possibly unrelated. We randomly select 575 pairs from them as our negative samples (label = 0). As there is no validated dataset for unrelated circRNA-Diseases pairs (negative samples), this approach allows us to have a balanced dataset and reduces the probability of having false negatives (i.e., CircRNA-Disease pairs that are really associated but whose associations have not been discovered yet) by a factor of $\frac{575}{{29761}} \cong 1.93\%$.

### Evaluate classification methods

**Fig. 2:**
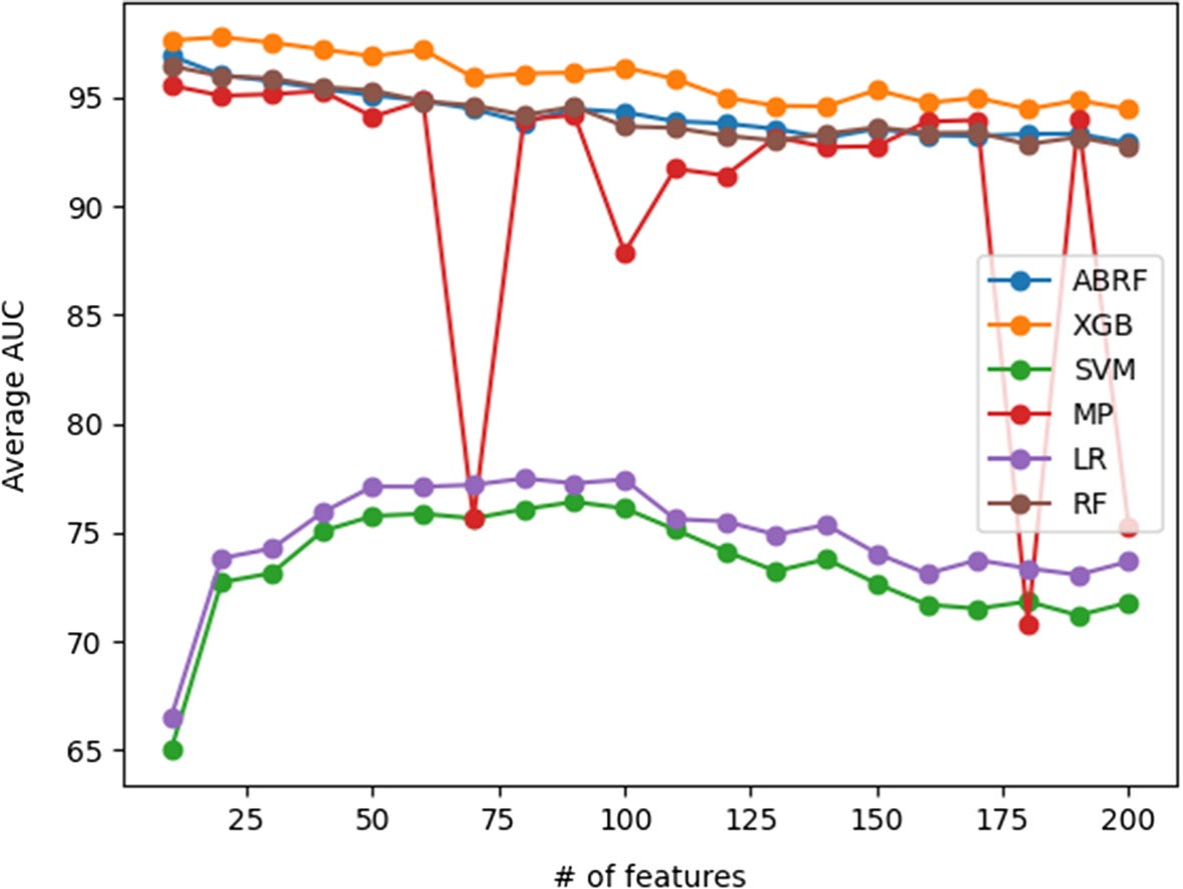
The average AUC of each classifier is based on the size of feature vectors extracted by DeepWalk

For each classifier, the evaluation statistics depend on the number of features of circRNAs and diseases in the CircRNA-Disease dataset fed to them as input. Therefore, using DeepWalk, we created a set of feature vectors with different vector sizes (a multiple of 10 ranging from 10 through 200). We obtained the classification results on the benchmark dataset for each classifier and found the optimal number of features in terms of AUC. Overall, the most accurate result we produced was achieved by the XGB and ABRF classifiers. Figure 2 shows how the average AUC of each classifier changes with the number of features extracted by DeepWalk. The optimal number of features for each classifier was considered for further evaluation.

Table 1 shows the values of the evaluation metrics for our six classifiers based on their optimal number of features. This table shows that SVM and LR have the minimum performance in our experiment with an average accuracy of 72 and 71, respectively. Overall, it seems that the boosting algorithms enjoy better performance compared with the others. Random forest shows the appropriate performance as well. In terms of accuracy, the random forest has the best result after XGBoost, but if we consider AUC, its effect is very close to AdaBoost. We employed AdaBoost to improve the random forest model results, but as you can see in the table, the results of these two approaches are very close. The XGBoost algorithm obtained the best result. We chose this algorithm as the classifier in our final pipeline. It is noteworthy, however, that the training time of the XGBoost classifier is by far longer than AdaBoost and random forest.

**Table 1:** The average values of the evaluation metrics in 5 folds for different classifiers based on their optimal number of features

Classifier (optimal feature vector size)	Acc (%)	F1 (%)	Pre (%)	Sen (%)	Spe (%)	AUC (%)
ABRF (10)	89.74	89.37	92.6	86.44	93.04	96.58
LR (80)	71.3	71.32	71.25	71.48	71.13	77.48
MP (10)	89.56	89.59	89.37	89.91	89.22	95.54
RF (10)	90.09	89.78	92.61	87.13	93.04	96.44
XGB (20)	92.09	92.078	92.36	91.82	92.35	97.77
SVM (90)	72.09	73.26	70.16	76.69	67.48	76.41

**Fig. 3:**
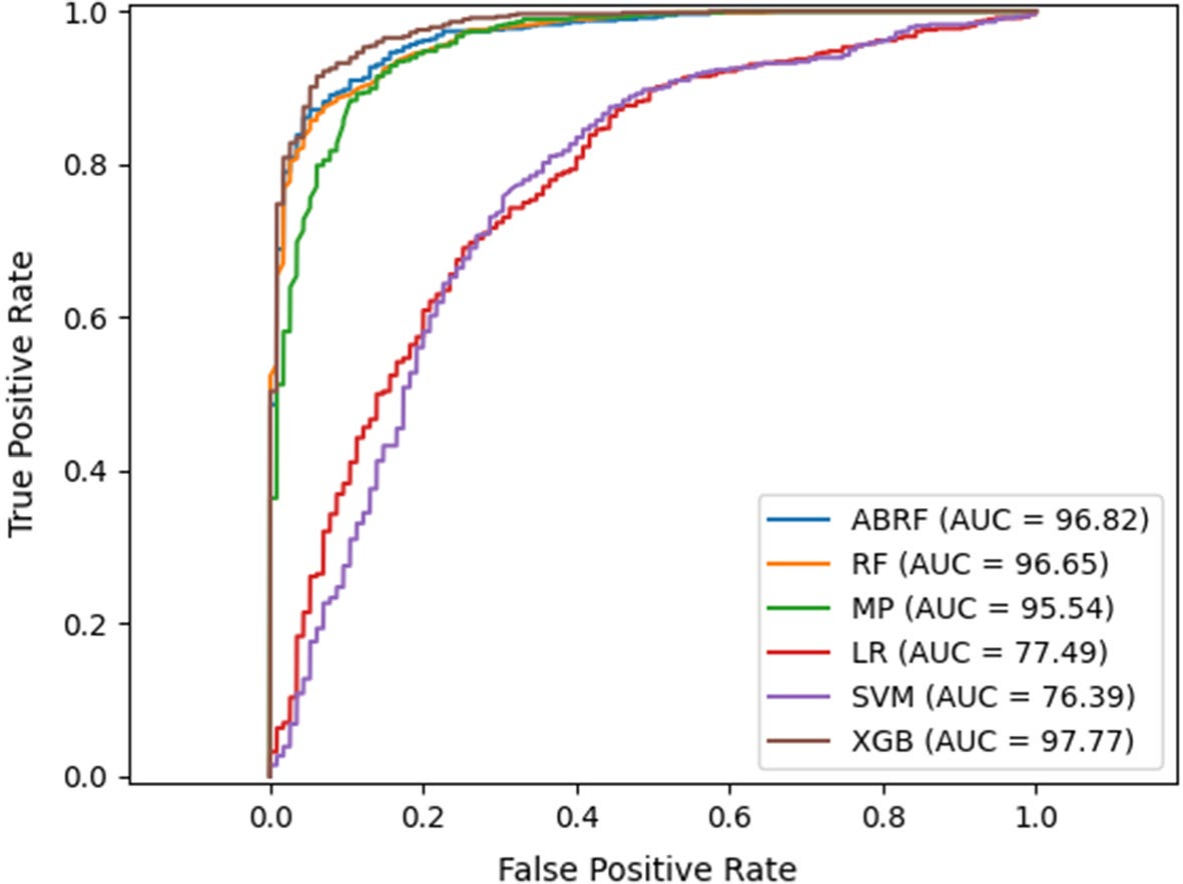
ROC curve and AUC based on the average values of 5 folds (the size of extracted feature vector with Deepwalk set to the optimum value for each classifier)

The permutation of the samples in the cross-validation folds was identical for all six classifiers. Figure 3 compares ROC curves of different classifiers for each fold of the data in the fivefold CV. Other algorithms outperformed SVM and logistic regression with an approximate gap of 20% in terms of all six metrics. Not to mention that the SVM took the longest training time of all models. The multilayer perceptron was superior to SVM and logistic regression but missed out on the others by about 1%. XGBoost had the highest AUC in 4 of the 5-folds of the dataset.

### Comparison with existing methods

We compared CircWalk with four state-of-the-art algorithms based on the benchmark dataset: DMFCDA (Deep Matrix Factorization CircRNA-Disease Association) [62], GCNCDA [61], GMNN2CD (Graph Markov Neural Network algorithm to predict unknown CircRNA–Disease associations) [63] and SIMCCDA (Speedup Inductive Matrix Completion for CircRNA-Disease Associations prediction) [64]. We applied a fivefold cross-validation approach to the benchmark dataset and trained each algorithm with its default parameters in each fold. The average value of the evaluation criteria in 5 folds was used to compare the algorithms.

Table 2 shows the evaluation process results for the selected algorithms based on the benchmark dataset. As shown in this table, CircWalk is the most outperforming algorithm in our experiment, and its average values for all evaluation metrics are more significant than 90%. After CircWalk, GMNN2CD is the best-performing algorithm among others. In terms of accuracy, this algorithm is the best in our experiment, but it has the lowest sensitivity compared with the other algorithms. GCNCDA is the most similar algorithm to our method among these comparison methods. Although this approach shows lower accuracy than CircWalk, it is more stable and shows approximately the same results in all folds. SIMCCDA has acceptable performance in all metrics except precision and F1. This algorithm accurately predicted the negative class (unassociated CircRNA-Disease pairs), but its true positive rate was meager.

**Table 2:** The average values of the evaluation metrics in 5 folds for different state-of-the-art algorithms based on the benchmark dataset

Algorithm	Acc (%)	F1 (%)	Pr (%)	Se (%)	Sp (%)	AUC (%)
CircWalk	92.09	92.08	92.36	91.83	92.35	97.77
DMFCDA	83.69	83.69	81.55	87.79	79.6	83.69
GCNCDA	74.52	74.9	73.79	76.17	72.87	82.72
SIMCCDA	83.36	16.4	9.1	84.54	83.34	73.3
GMNN2CD	99.09	85.52	72.63	63.36	99.78	96.69

**Fig. 4:**
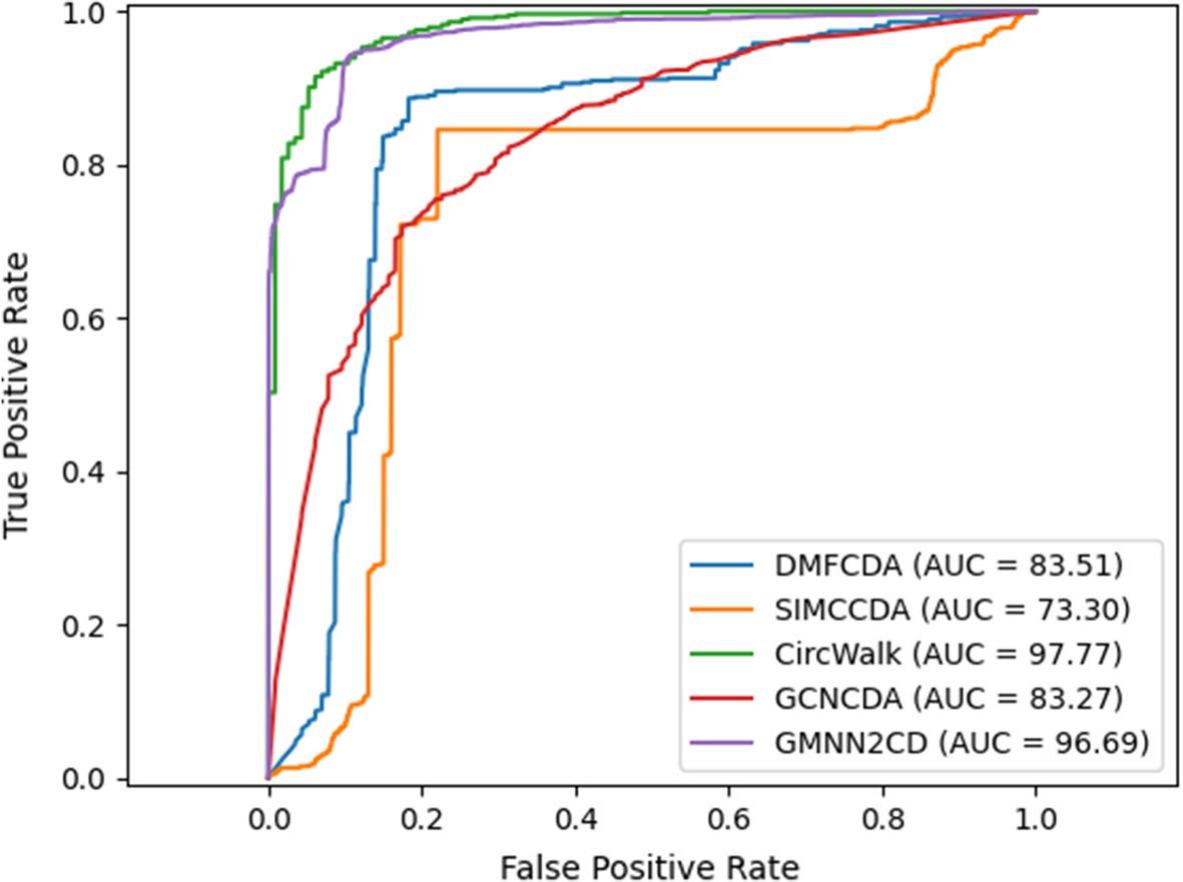
ROC curve and AUC based on the average values of 5 folds for different algorithms compared with our method

Figure 4 compares the ROC curve of each algorithm in each fold of the validation. As shown in this figure, CircWalk obtained an AUC of more than 96% (about 97% on average). GCNCDA and DMFCDA have almost the same results, and SIMCCDA has the poorest results in our experiment (because of its low true positive rate).

### Case study

This step aims to evaluate the performance of CircWalk in the prediction of novel CircRNA-Disease associations in some selected common diseases. To this end, we selected three common cancers (lung, gastric, and colorectal) that are the target of many circRNA-related kinds of research. We train our model on the feature vectors of the positive pairs and a third of the negative pairs. As we pointed out earlier, the negative pairs (i.e., associations) are a subset of unverified CircRNA-Disease associations, which means there may be positive associations. As a result, we decided to train our model on a few negative pairs as possible to reduce learning from these false negatives. However, we could not wholly omit them as there must be at least two classes in the dataset for XGBoost to be trained on it. Then, we list all CircRNA-Disease pairs whose circRNA is present in our initial CircRNA-Disease dataset and whose disease is one of the three diseases we selected in this part. After that, filter out the CircRNA-Disease pairs present in the data, which our model was trained on in this part. We give this list of CircRNA-Disease associations as input to our trained model. Instead of labeling them as positive (1) or negative (0), we use our model to calculate the probability of association in each pair. Finally, for each disease, we find the circRNAs that are most likely to be associated with that disease and investigate the existing literature in PubMed to check if empirical studies have already confirmed that CircRNA-Disease association. Table 3 shows the result of this investigation.

**Table 3:** Predicted CircRNA-Disease relations with the highest probability for some selected diseases

Disease	circRNA	Probability	Related article (PMID)
Lung cancer	hsa_circ_0007534	0.996	30017736
	hsa_circ_0001946	0.995	31249811
	hsa_circ_0002874	0.992	33612481
	hsa_circ_0014130	0.991	29440731, 31241217, 31818066, 32060230, 32616621, 34349347
	hsa_circ_0002702	0.990	32962802
	hsa_circ_0007874	0.988	30975029
	hsa_circ_0074930	0.985	32962802
	hsa_circ_0086414	0.983	30777071
	hsa_circ_0079530	0.972	29689350
	hsa_circ_0007385	0.972	29372377, 32602212, 32666646
	hsa_circ_0016760	0.968	29440731
	hsa_circ_0012673	0.960	29366790, 32141553
	hsa_circ_0067934	0.954	33832139
	hsa_circ_0000567	0.950	32328186, 33768996, 34435479
	hsa_circ_0072088	0.941	32308427, 34135596
	hsa_circ_0001727	0.934	32010565
	hsa_circ_0008305	0.901	30261900
Gastric cancer	hsa_circ_0001313	0.999	32253030
	hsa_circ_0004771	0.998	29098316
	hsa_circ_0002874	0.998	34388244
	hsa_circ_0000615	0.998	34049561
	hsa_circ_0006404	0.977	32445925
	hsa_circ_0001982	0.977	33000178
	hsa_circ_0032683	0.910	33449227
	hsa_circ_0014130	0.819	32190005
Colorectal cancer	hsa_circ_0006054	0.995	30585259
	hsa_circ_0000745	0.990	28974900
	hsa_circ_0044556	0.989	32884449
	hsa_circ_0005075	0.964	31081084, 31476947, 34015582
	hsa_circ_0040809	0.958	34438465
	hsa_circ_0004771	0.945	31737058, 32419229
	hsa_circ_0007874	0.924	32419229
	hsa_circ_0080210	0.914	34222420

As shown in Table 3, all the predicted pairs (except gastric cancer) had a probability of over 90%. There is much experimentally validated evidence in the results of this step. For instance, CircWalk predicted an association between hsa_circ_0001313 and gastric cancer with a probability of almost 100%. Based on a recent study by Zhang et al. [65], this circRNA is a vital regulator of drug resistance in gastric cancer. CircRNA hsa_circ_0007534 (predicted by a probability of 99.6%) is an essential oncogene in lung cancer related to cancer cell proliferation and apoptosis [66].

Another example is the association between hsa_circ_0044556 and colorectal cancer (predicted by a probability of 98.9%). Knocking down this circRNA prevents proliferation, migration, and invasion of colorectal cancer cells [67]. These results represent the power of CircWalk to predict truly novel CircRNA-Disease associations.

## Discussion

This study tried to integrate multiple data from multiple resources about genes and disease interactions to predict more significant CircRNA-Disease associations. Although biological data generation technologies have been advanced in recent years, this data type is primarily incomplete and has false positives. Data Integration can be a helpful approach to reducing noise and false positives. Biological events are closely related and work as a system on the plus side. The cause of many disorders in the human body can only be explained using this systematic view of cellular processes. Therefore, the main idea we have to solve the problem of our study is to integrate multiple data into a complex network and try to find associations between circRNAs and diseases through the network's features. Another critical point in our approach involves the concept of the ceRNA hypothesis and the miRNA sponge effect of circRNAs to predict their associations with diseases. The results of our study demonstrated that this point of view could help predict CircRNA-Disease associations more accurately.

One of the most challenging steps in our study was preparing the data. Each dataset uses unique identifiers for circRNAs, and converting these identifiers sometimes can be impossible. So, we missed some information in our data because we couldn’t convert and match the identifiers of some circRNAs in multiple datasets. This limitation can affect the results of our algorithm, and defining a standard for naming this type of RNA and creating a comprehensive database is needed. Another challenge that can significantly affect the result of algorithms is the lack of validated negative classes (un-associated pairs) for the CircRNA-Disease associations. As we mentioned in previous sections, we generated the negative class by randomly selecting circRNAs and unassociated diseases in our data. But there is no guarantee that there is no association between these selected negative pairs. This misinformation can affect the learning process of the classifiers and lead to generating inappropriate models. Consequently, creating standardized datasets and benchmarks to validate the models is one of the ideal approaches for the future of this field of study.

It is necessary to keep in mind that the sponging effect of circRNAs is not the only biological aspect that can help predict their association with a disease. Some other biological information can solve the problem in future work. For instance, their expression data, their exonic or intronic structure, the miRNA response elements information related to their sequence, and any other information about their structure and function can help associate them with a disease, provided that the related data be accessible. Furthermore, using novel machine learning approaches such as deep learning and graph convolutional neural networks can integrate multiple data and extract meaningful features in the study's next step.

The presented algorithm can be used to predict miRNA-Diseases and lncRNA-Disease associations. To this end, we need to extract feature vectors of miRNAs and lncRNAs instead of circRNAs. Also, miRNA-miRNA, lncRNA-lncRNA, and lncRNA-Disease associations should be added to the data.

## Supplementary Information

**Additional file 1: Table S1.** Classifiers hyperparameters.

## Data Availability

The datasets generated or analyzed during the current study are available in the CircWalk repository, at https://github.com/bcb-sut/CircWalk, with the source code. As for the raw data, the twelve datasets we used can be found at the following web addresses, respectively: Circ2Disease at http://bioinformatics.zju.edu.cn/Circ2Disease, CircR2Disease at http://bioinfo.snnu.edu.cn/CircR2Disease, CTD at https://ctdbase.org, circAtlas at http://circatlas.biols.ac.cn/, circBase at http://www.circbase.org/, RAID at http://www.rna-society.org/404.shtml, starBase at http://www.sysu.edu.cn/403.html, HMDD at http://www.cuilab.cn/hmdd, miR2Disease at http://www.mir2disease.org/, miRTarBase at https://mirtarbase.cuhk.edu.cn/~miRTarBase/miRTarBase_2022/php/index.php, DisGeNET at https://www.disgenet.org/, and MeSH at https://www.nlm.nih.gov/mesh/meshhome.html.
