# Supplementary material for: CircWalk: a novel approach to predict CircRNA-disease association based on heterogeneous network representation learning
Source: BMC Bioinformatics. 2022 Aug 11;23:331. doi: 10.1186/s12859-022-04883-9 (PMC9367077; doi:10.1186/s12859-022-04883-9)
Supplement: Supplementary file 1 — Additional file 1: Table S1. Classifiers hyperparameters. [file 12859_2022_4883_MOESM1_ESM.docx]

Supplementary Table 1. Classifiers hyperparameters.

| Method | Hyperparameters |
| --- | --- |
| SVM | $kernel=\text{linear}\text{}$ $C=5$ $probability=True$ |
| LR | $C=10$ $\max\_iter=1000$ |
| RF | $\max\_depth=\frac{embedding size}{3}$ |
| ABRF | $\max depth=10$ $learning rate={10}^{-2}$ |
| MP | $solver=^{'}lbfgs^{'}$  $alpha=0.01$  $hidden layer sizes=\left( 10,2 \right)$  $\max\_iter=1000$  $random state=1$ |
| XGB | $eta: 0.3$ $max\_depth: 7$ $objective:'multi:softprob^{'}$ $num_{class}: 2$ $steps = 20$ $num\_round = 64$ |
